# Predictors of Food Insecurity Before and During COVID-19 Among Middle-Aged and Older Adults

**DOI:** 10.1093/geroni/igab046.3420

**Published:** 2021-12-17

**Authors:** Emily Nicklett, Jianjia Cheng

**Affiliations:** 1 The University of Texas at San Antonio, San Antonio, Texas, United States; 2 School of Public Health, Ann Arbor, Michigan, United States

## Abstract

The indirect impact of COVID-19 on food security of middle aged and older adults is not well understood. This study examines changes in risk factors for food security from 2018-2020 in a population-based sample. Using data from the Health and Retirement Study (2018 and 2020 waves), we utilized generalized estimating equations (GEE) with repeated measures to examine factors associated with food insecurity among US adults aged 50 and older (n=3170) before COVID-19 and since COVID-19. The prevalence of food insecurity doubled from 2018 (4.83%) to 2020 (9.54%). In multivariate analyses, the population-averaged odds of experiencing food insecurity was 81% higher in 2020 compared to 2018. Other factors significantly associated with higher odds of food insecurity included being female (OR: 1.29), Black (OR: 1.46), lowest quintile for wealth (OR: 1.82), not working due to a disability (OR: 3.29), renting (OR: 2.04), greater IADL limitations (OR: 1.32), and greater number of chronic illness comorbidities (OR: 1.14). Factors significantly associated with lower odds of food insecurity included older age (65-74: OR: 0.73; 75+: OR: 0.56) and being above the median income level (OR: 0.47). Partnership status, education level, and ADL limitations were not significantly associated with the population-averaged odds of experiencing food insecurity. This study identified factors related to food insecurity among a community-dwelling sample of middle aged and older adults in the U.S. Future research should examine the impact of policies and intervention strategies to address the disproportionate impact of COVID-19 on populations at increased risk of experiencing food insecurity.

